# Endovascular treatment of symptomatic vasospasm and delayed cerebral ischemia after aneurysmal subarachnoid hemorrhage – a systematic review and meta-analysis

**DOI:** 10.1007/s00234-026-03986-x

**Published:** 2026-03-31

**Authors:** Michael Veldeman, Tobias Rossmann, Roel Haeren, Hanna Schenck, Rahul Raj, Charlotte S. Weyland

**Affiliations:** 1 Department of Neurosurgery, Universitätsklinikum Aachen, Aachen, Germany; 2 Department of Neurosurgery, Johannes Kepler University of Linz, Linz, Austria; 3 Department of Neurosurgery, Maastricht University Medical Centre, Maastricht, Netherlands; 4 Department of Neurosurgery, Helsinki University Hospital, Helsinki, Finland; 5 Department of Diagnostic and Interventional Neuroradiology, Universitätsklinikum Aachen, Aachen, Germany

**Keywords:** Aneurysmal subarachnoid hemorrhage, Intra-arterial spasmolysis, Angioplasty, Delayed cerebral ischemia, Endovascular treatment, Cerebral vasospasm

## Abstract

**Background:**

Endovascular rescue therapy, including intra-arterial spasmolysis and balloon angioplasty, is widely used for symptomatic vasospasm and delayed cerebral ischemia (DCI) after aneurysmal subarachnoid hemorrhage (aSAH), but its impact on functional recovery is unclear. We systematically reviewed evidence on patient-centered outcomes, including effects by follow-up time, intervention type, and clinical severity.

**Methods:**

PubMed, EMBASE, and Web of Science were searched (2000–2024; updated July 2025). Prospective and retrospective studies of adults with aSAH receiving endovascular treatment for symptomatic vasospasm/DCI were included. Data extraction followed a PICO framework, and quality was assessed using the Newcastle–Ottawa Scale. Due to inconsistent reporting of angiographic resolution and DCI-related infarction, quantitative synthesis focused on dichotomized favorable functional outcome. Single-arm proportions were pooled using random-effects generalized linear mixed models with prespecified subgroup analyses.

**Results:**

Thirty-nine studies (1,627 patients) were included; 38 contributed to pooled analysis. The proportion of favorable functional outcome was 0.55 (95% CI 0.50–0.61), with substantial heterogeneity (I² ≈ 71%). Subgroup analyses by follow-up duration, intervention modality, and baseline severity showed no significant differences. The two randomized trials reported conflicting results and had limited follow-up. Safety reporting varied but was generally acceptable for pharmacologic spasmolysis; some series suggested higher complication rates with mechanical interventions.

**Conclusion:**

Approximately half of patients treated with endovascular rescue for symptomatic vasospasm/DCI achieve a favorable functional outcome. However, marked heterogeneity and predominantly observational evidence limit conclusions regarding effectiveness. Standardized, adequately powered randomized trials are needed to clarify the role of endovascular rescue after aSAH.

**Supplementary Information:**

The online version contains supplementary material available at 10.1007/s00234-026-03986-x.

## Key messages

*****What is already known on this topic***:**

- Endovascular rescue for delayed cerebral ischemia is widely used, but its effect on functional outcomes remains unclear due to predominantly small and heterogeneous observational studies.

*****What this study adds***:**

- Across 39 studies, about half of patients achieved favorable functional outcomes after endovascular treatment, with no clear differences by intervention type, follow-up duration, or baseline severity.

*****How this study might affect research, practice or policy***:**

- These findings highlight persistent uncertainty regarding effectiveness and underscore the need for standardized, adequately powered randomized trials to define the role of endovascular rescue in clinical practice.

**Supplementary Information:**

The online version contains supplementary material available at 10.1007/s00234-026-03986-x.

## Introduction

### Rationale

Delayed cerebral ischemia (DCI) remains the predominant complication contributing to poor neurological outcome following aneurysmal subarachnoid hemorrhage (aSAH) [1]. If left undiagnosed or when unsuccessfully treated, it may result in DCI-related infarction. Aside from the prophylactic use of oral nimodipine, no therapeutic intervention to date has consistently demonstrated a reduction in DCI incidence or severity nor contributed to an improvement in long-term clinical outcomes [2, 3].

Cumulative observational data suggest that induced hypertension, achieved through vasopressor infusion, serves as a first-line intervention upon diagnosis of DCI. However, a randomized controlled trial (RCT) evaluating this approach failed to demonstrate clinical benefit and was terminated early due to hypertension-related complications [4]. This study has faced criticism for employing excessively high blood pressure targets, which likely contributed to the elevated complication rate. Moreover, the trial was underpowered to evaluate functional outcomes meaningfully.

While our understanding of DCI pathophysiology has evolved beyond a sole focus on angiographic large-vessel vasospasm, this phenomenon is still believed to represent the final common pathway leading to ischemic decompensation in vulnerable brain tissue. Contributing mechanisms include impaired cerebral autoregulation, disrupted neurovascular coupling, microthrombosis, microvasospasm, as well as cortical spreading depolarizations and ischemia [5].

Notably, pharmacologic interventions that successfully reduced angiographic vasospasm have consistently failed to improve functional outcomes, highlighting the complexity of DCI pathogenesis [6]. In recent years, advances in endovascular microcatheter technology and the availability of intra-arterial antithrombotic, vasodilatory agents, and intra-vascular devices [7] have enabled the emergence of endovascular treatment strategies for DCI. Intra-arterial spasmolysis, via the infusion of vasodilators, has shown promise in resolving diffuse and distal vasospasm [8], while transluminal balloon angioplasty may induce more sustained vessel dilation, albeit limited to focal, proximal vasospasm [9].

Both techniques have been reported across numerous observational studies. To date, only two small randomized controlled trials have compared endovascular therapy with conservative management, but differences in study design and outcome evaluation have resulted in inconclusive and conflicting findings [10, 11].

### Objectives

This systematic review and meta-analysis aim to synthesize the current evidence regarding the efficacy of endovascular treatment for DCI following aSAH, specifically in (1) improving functional clinical outcome (2) and reducing DCI-related infraction.

## Methods

### Protocol and registration

The review protocol was developed in September 2022 and prospectively registered in the Research Registry [REDACTED FOR REVIEW] in October 2022. It defined eligibility criteria, the search time frame, and a trial evaluation checklist. Reporting followed PRISMA guidelines and Cochrane methodological standards.

## Eligibility criteria

We included prospective or retrospective studies (≥ 5 patients) assessing intra-arterial treatment for delayed cerebral ischemia (DCI) after subarachnoid hemorrhage, published between January 2000 and July 2025. Eligible interventions included intra-arterial vasodilator infusion or mechanical angioplasty (e.g., balloon or Comaneci device) in adults (≥ 18 years). For the purposes of this review, ‘symptomatic vasospasm’ and ‘delayed cerebral ischemia’ were treated as overlapping indications reflecting real-world heterogeneity in the included literature. Where reported, DCI was defined according to established clinical criteria, namely, the occurrence of a new focal neurological deficit and/or a decline in level of consciousness not attributable to other causes [12], with or without supportive findings on imaging (CT perfusion, MRI-DWI), transcranial Doppler, or angiography. However, a substantial proportion of included studies used angiographic vasospasm alone, with or without clinical correlate, as the primary indication for endovascular treatment. This diagnostic heterogeneity was noted during data extraction and is discussed as a limitation. Feasibility, safety, or technical reports, protocols, and series < 5 patients were excluded. Studies had to report ≥ 1 of the following: angiographic vasospasm resolution, radiologic DCI (CT or TCD), DCI-related infarction, or clinical outcomes. Because few studies included control groups, their presence was not mandatory but was noted for pooled analyses when outcomes were comparable.

Information Sources and Search Strategy.

PubMed, EMBASE, and Web of Science were searched in January 2023 and updated in July 2025 using combinations of *cerebral vasospasm*, *delayed cerebral ischemia*, *subarachnoid hemorrhage*, *spasmolysis*, and *angioplasty*, including British/American spelling variants. Reference lists of included studies were screened manually. Searches were restricted to English, German, French, or Dutch; other languages were considered if translation was feasible.

## Data collection

Two reviewers [REDACTED FOR REVIEW], independently screened studies in Rayyan (www.rayyan.ai) and extracted data using a standardized PICO-based template. Rayyan was only used to share papers and extracted data among authors. No generative artificial intelligence was used for data extraction. Recorded variables included demographics, hemorrhage severity, indication and type of endovascular therapy, prior medical management, definitions of DCI, complications, and clinical and radiologic outcomes. Discrepancies were resolved by consensus.

### Risk of bias

Non-randomized studies were appraised with the Newcastle–Ottawa Scale (NOS), scoring up to eight points for selection, comparability, and outcome assessment. Scores were converted to qualitative categories (good, fair, poor) per AHRQ guidance. Two reviewers rated studies independently, resolving disagreements by discussion.

### Data analysis

Meta-analyses of single-arm proportions for favorable functional outcome were conducted in R (v4.4.0; *meta* package). Conventional cutoffs were applied (mRS 0–2, GOS 4–5). Random-effects generalized linear mixed models were used for primary and subgroup analyses (by follow-up duration, intervention type, and clinical severity), with τ², I², and Cochran’s Q quantifying heterogeneity. Forest plots display study estimates, 95% CIs, and inverse-variance weights. Funnel plots and Egger’s regression tested small-study effects when ≥ 10 studies were available. Statistical significance was set at two-sided *p* < 0.05.

## Results

### Study selection

**Fig. 1:**
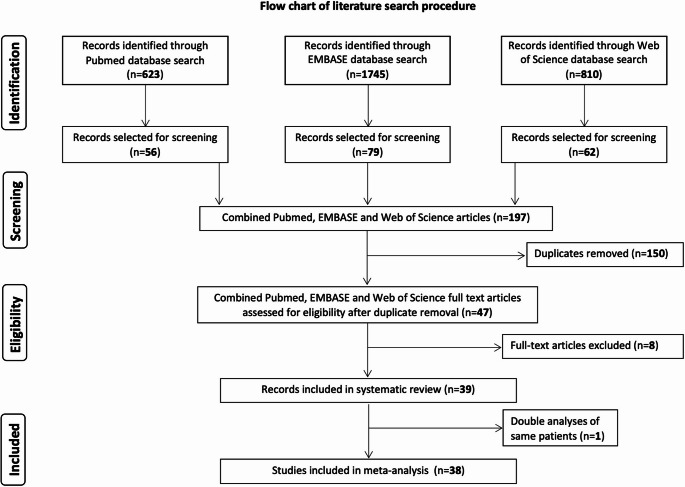
PRISMA flow diagram of literature search and study selection process. Systematic literature search across PubMed (*n* = 623), EMBASE (*n* = 1745), and Web of Science (*n* = 810) databases. After duplicate removal and successive screening, 39 studies were included in the systematic review. Two studies with overlapping patient cohorts from the same center were identified [9, 13]; only one was included in the meta-analysis to avoid double-counting patients, resulting in 38 studies for quantitative analysis

Our search strategy identified 3,178 studies across three databases (PubMed, EMBASE, and Web of Science), of which 197 articles remained before duplicate removal (*n* = 150). Following title and abstract screening, 47 full-text articles were assessed for eligibility. Eight manuscripts were excluded as they did not fulfill the defined inclusion criteria after full-text analysis. Finally, 39 studies met the eligibility criteria and were included in the systematic review, with 38 studies contributing data to the meta-analysis. One study was excluded to avoid double-counting, as it entailed a secondary analysis of the same patient cohorts. The literature search procedure is depicted in Fig. 1. All included studies and their characteristics are summarized in Suppl. Table 1.

### Study characteristics

Of the 39 studies included in this systematic review, 27 were retrospective cohort studies (r-co) [8, 14–39], five were case series (cs) [40–44], five were prospective cohort studies (p-co) [9, 13, 45–47], and two were randomized controlled trials (RCTs) [10, 11]. Across all included studies, a total of 1,627 patients received endovascular treatment for delayed cerebral ischemia. The number of patients per study ranged from 5 to 202. A control group of some sort, was present in 13 studies [8, 10, 11, 15, 17, 19, 21, 24, 27, 31, 38, 41, 44].

### Risk of bias within and across studies

The methodological quality of the included studies, as assessed by the Newcastle–Ottawa Scale (NOS), varied considerably. Of the 39 studies, 11 were rated as having poor methodological quality, 20 as moderate, and only 8 studies were classified as high quality, according to the Agency for Healthcare Research and Quality (AHRQ) conversion standards. The average NOS score across all studies was 5.15 out of a possible 9, indicating a generally moderate to high risk of bias across the dataset.

The most frequent sources of bias were related to comparability between groups, which was inadequately addressed in 33 studies, and adequacy of follow-up, which was either unclear or insufficient in 23 studies. Selection bias was also common: 20 studies failed to report or implement appropriate selection of the non-exposed cohort, and 16 studies demonstrated limited representativeness of the exposed population. In contrast, outcome assessment and ascertainment of exposure were generally well reported, though still lacking in 6 and 5 studies, respectively. Taken together, the overall methodological limitations, especially in non-randomized studies, highlight the need for caution in interpreting pooled results. Detailed results of our interpretation and implementation of the RoB via the NOS tool are provided as Suppl. Table 2.

### Results of individual studies

#### Randomized trials

To date, only two small RCTs have directly compared endovascular therapy to conservative medical management. A German trial randomized 34 patients with DCI (defined by perfusion deficit in perfusion MRI scanning), allocating 16 to receive endovascular treatment and 18 to standard care [10]. The trial was terminated prematurely due to a higher incidence of unfavorable outcomes, defined by the modified Rankin Scale (mRS) at six months, in the intervention arm. This cohort had not received prior induced hypertension, raising questions about treatment sequencing and patient selection.

In a second trial by Yindeedej et al., 68 patients were enrolled, with 36 undergoing intra-arterial nimodipine (IAN) treatment [11]. This group demonstrated early neurological improvement, particularly in consciousness and motor function as measured by the Glasgow Coma Scale. However, the trial only included patients with an evaluable neurological examination, the clinical criteria for IAN administration were imprecise, and no follow-up beyond hospital discharge was provided.

### Prospective cohort studies

Small prospective cohorts showed short-term angiographic or physiologic responses with variable clinical impact. In 18 aSAH patients, balloon angioplasty or IAN produced transient perfusion and angiographic improvement [45]. In 10 patients with abnormal invasive neuromonitoring, intra-arterial papaverine improved cerebral metabolism but the effect was short-lived [46]. Among 25 patients with MRI-confirmed DCI, nimodipine spasmolysis or angioplasty was associated with a lower rate of DCI-related infarction [47]. In a study by Weiss et al., about half of endovascularly treated patients reached functional independence at one year with low procedural complication rates. Bedside continuation of spasmolysis often increased vasopressor requirements, however, in this study, continuous spasmolysis in selected patients showed low complication rates and physiologic improvement, and 33% achieved favorable outcome at 3 months [13].

### Retrospective cohort studies

Across retrospective cohorts, endovascular rescue commonly produced angiographic improvement, while clinical benefit varied. For example, Albrecht et al. reported 42.8% favorable outcome at 6 months and a low procedural complication rate of 4% across 241 interventions, with no ischemic or thromboembolic events recorded [15]. Early and frequent endovascular therapy was associated with lower infarction rates and better functional outcomes in a large pooled cohort analysis, although residual confounding is possible (Jabbarli 2019) [8].

Intra-arterial nimodipine (IAN) was the most frequently used regimen, occasionally continued bedside (CIAN) at the intensive care unit (ICU). Several cohorts described effective vasodilation and, in places, fewer infarcts or better outcomes, while others noted limited translation of angiographic gains into clinical benefit [17, 18, 21, 23, 24, 32]. Milrinone showed vasodilatory effects, particularly at higher doses and in combination with nimodipine, but clinical outcomes were inconsistently reported [20]. Papaverine often produced stronger angiographic dilation than nimodipine without clear clinical advantage [26]; bedside continuation of spasmolysis in the ICU was linked to improved outcomes in one historical comparison [41].

Angioplasty was widely used as rescue or adjunct therapy. One cohort found no evidence of delayed arterial re-narrowing after angioplasty [33]. Device-based strategies, including stent retrievers with nicardipine and permanent stents, were described as technically feasible in refractory cases, but evidence remains limited and highly selected [27, 30]).

Standardized perfusion imaging to guide rescue was associated with fewer infarcts and better function in one cohort (Mielke [31]). Others noted discordance between immediate angiographic response and downstream infarction or clinical outcome, and in some cases greater vasodilation on delayed angiography than immediately post-procedure [34, 36, 39]).

Most series reported acceptable safety profiles, however, the reporting and definition of complications were heterogenous. High complication rates and a 6% mortality were noted in one cohort, advising caution in interpretation without controls [25]. Continuous IAN was generally safe yet carried extracranial risks, such as cardiac problems and infections [25]). Physiologic improvements such as normalization of ICP and brain tissue oxygenation were observed during intra-arterial papaverine in patients failing medical therapy [37].

### Case series

Small single-center series consistently report angiographic improvement after endovascular rescue. For IAN, a 42-patient series with 101 infusions showed 82.2% angiographic response, 68.3% immediate clinical improvement, 21.4% vasospasm-related infarction, and no drug-related complications [43]. Other reports describe reversal of angiographic spasm with clinical benefit varying by timing, vasospasm burden, and number of procedures [40]. Continuous IAN was associated with better outcomes and fewer strokes versus a historical control [41]. Additional series judged IAN effective and safe in selected patients while calling for prospective confirmation [42].

In a 69-patient comparison, intra-arterial papaverine, balloon angioplasty, or both improved angiography without differences in short-term neurological improvement; effects did not correlate consistently with TCD and showed no clear time-dependence [44].

### Adverse events

A tabular summary of reported adverse events across all 39 studies, stratified by intervention type, is provided in Suppl. Table 3. Adverse event reporting was heterogeneous: 9 studies did not report complications at all, and definitions, ascertainment methods, and the denominator (patients *versus* number of interventions) used varied considerably across the remainder, precluding quantitative pooling of safety data.

### Synthesis of results - meta-analysis

We planned to pool three outcomes: resolution of angiographic vasospasm, DCI-related infarction, and dichotomized functional outcome. Reporting of the first two was too sparse or inconsistently defined to permit pooling, and too few studies included a control group for comparative meta-analysis. We therefore performed single-arm meta-analyses of proportions for dichotomized functional outcome and present pooled estimates overall and stratified by follow-up time (≤ 90 vs. > 90 days), intervention type (intra-arterial spasmolysis, balloon angioplasty, combined), and clinical severity (≥ 40% vs. < 40% poor grade).

### Stratified by follow up time

**Fig. 2:**
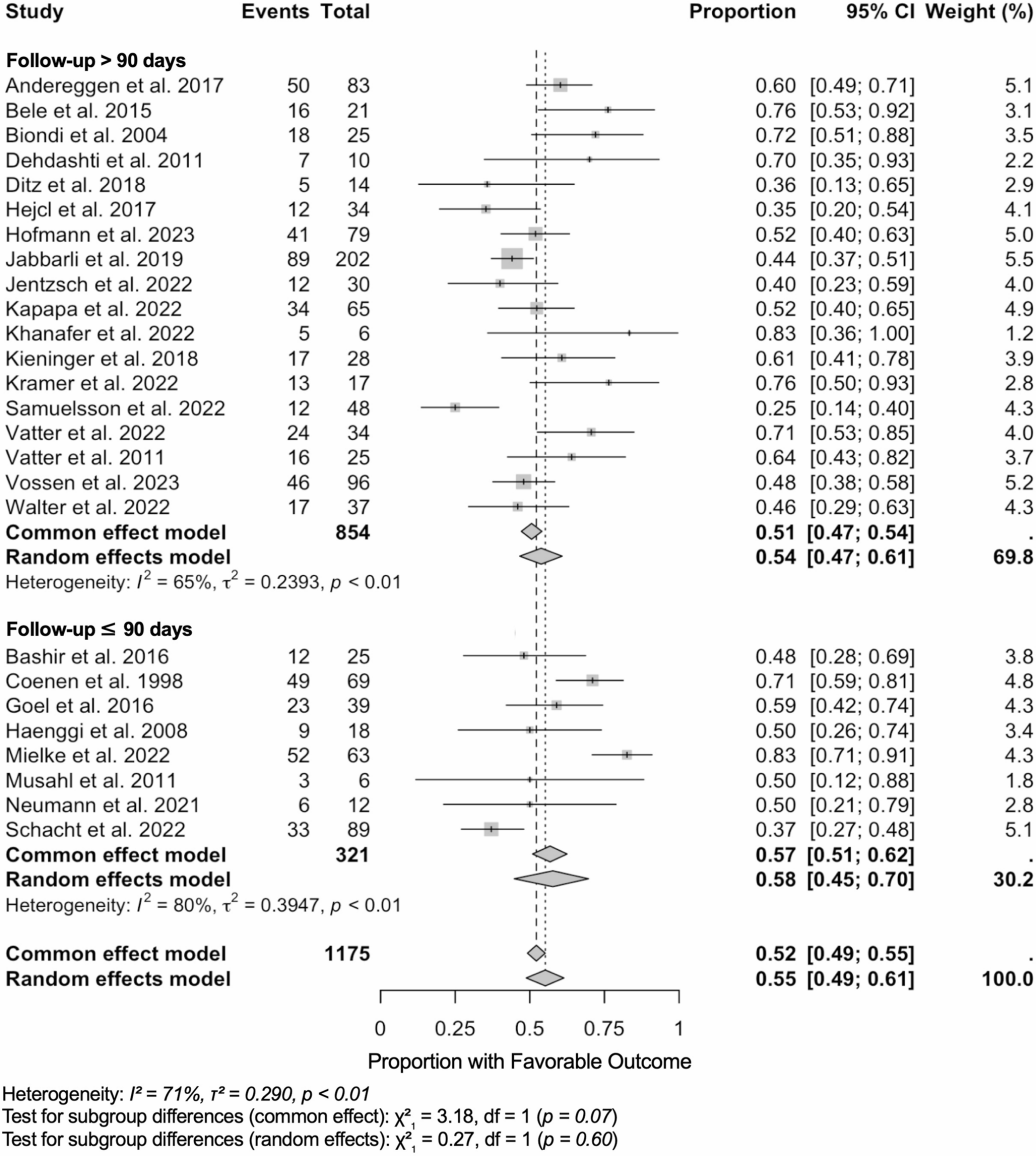
Forest plot of favorable functional outcomes following endovascular treatment for delayed cerebral ischemia, stratified by follow-up duration. Forest plot showing proportions of patients achieving favorable functional outcomes after endovascular treatment for delayed cerebral ischemia (DCI), stratified by follow-up duration. Each study is represented by a square with horizontal lines indicating 95% confidence intervals (CI). Square size is proportional to study weight in the meta-analysis. Studies are grouped by follow-up duration: long-term follow-up (> 90 days) and short-term follow-up (≤ 90 days). Pooled estimates are shown as diamonds for each subgroup and overall. The common effect model assumes homogeneous treatment effects across studies, while the random effects model accounts for between-study heterogeneity. Study weights are calculated using the inverse variance method, where larger studies and those with more precise estimates receive greater weight. Heterogeneity statistics: I² represents the percentage of variability due to heterogeneity rather than sampling error; τ² (tau-squared) quantifies between-study variance; Q-test p-values assess statistical significance of heterogeneity. Subgroup differences were tested using chi-square tests for both common and random effects models. The vertical dashed line at 0.5 represents the null hypothesis of no treatment benefit (50% favorable outcome rate). Results favoring treatment efficacy appear to the right of this line. A total of 26 studies with 1,175 patients and 621 events were included in this analysis. CI, confidence interval; df, degrees of freedom; I^2^, I square statistic providing the percentage of variation across studies that is due to heterogeneity; τ² (tau-squared statistic representing between-study variance in the random effects meta-analysis; χ², (chi-squared) statistic refers to the test statistics for subgroup differences

We synthesized single-arm proportions of favorable functional outcome on the logit scale using inverse-variance weighting and fitted both common-effect and random-effects models, reporting back-transformed proportions. These results are presented as a forest plot (Fig. 2). Twenty-seven studies (*N* = 1,175) were included. Overall, the pooled proportion was 0.55 (95% CI 0.49–0.61) under the random-effects model and 0.52 (0.49–0.55) under the common-effect model, with substantial heterogeneity (I² = 71%, τ² = 0.290, *p* < 0.01). By follow-up, ≤ 90 days (9 studies; *N* = 321) yielded 0.58 (0.45–0.70) with high heterogeneity (I² = 80%, τ² = 0.395), and > 90 days (18 studies; *N* = 854) yielded 0.54 (0.47–0.61) with moderate heterogeneity (I² = 65%, τ² = 0.239). Tests for subgroup differences were not statistically significant (random-effects χ² = 0.27, *p* = 0.60; common-effect χ² = 3.18, *p* = 0.07). Most precision came from the > 90-day subgroup, which contributed about 70% of the total weight.

### Stratified by endovascular method

**Fig. 3:**
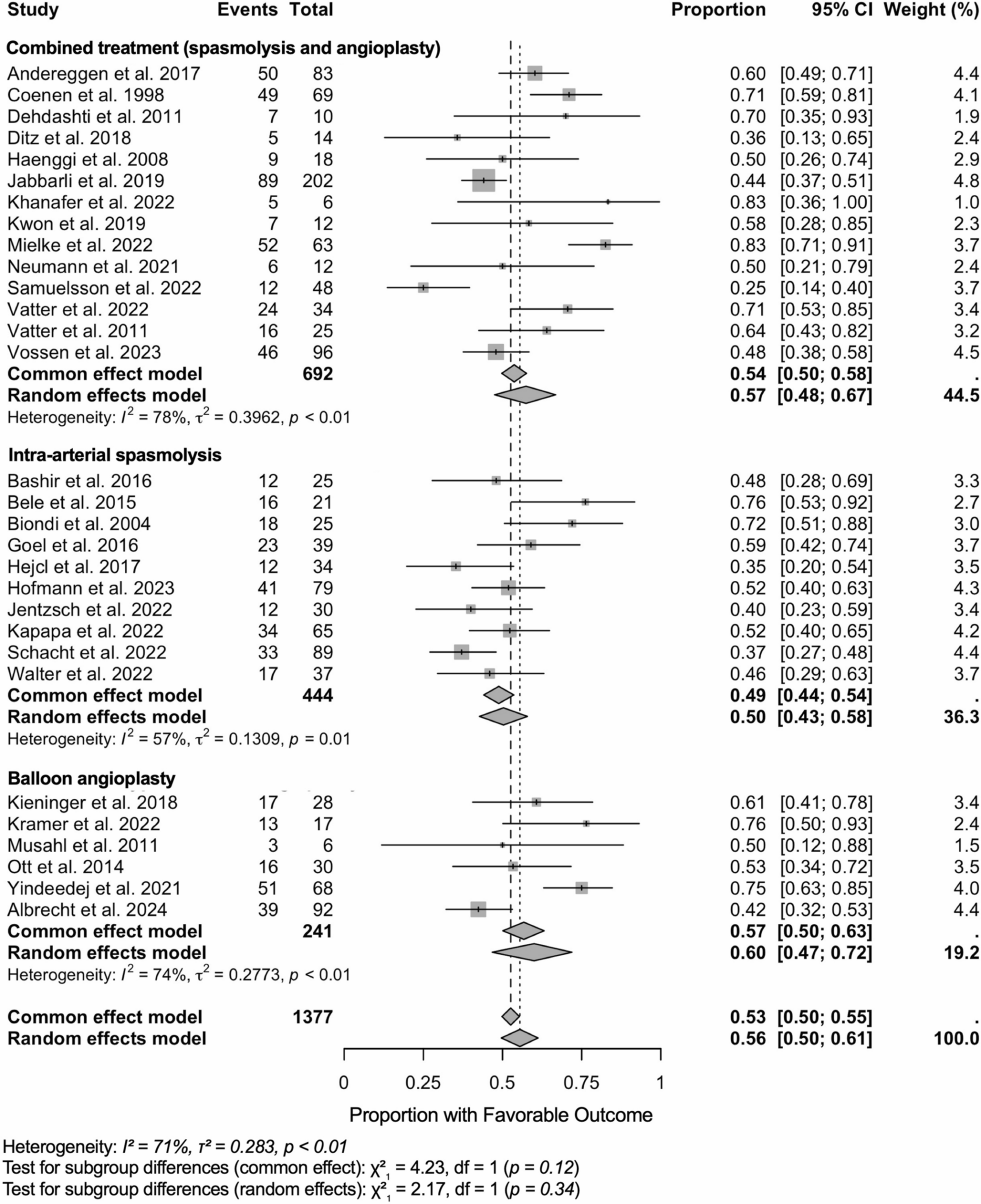
Forest plot of favorable functional outcomes following endovascular treatment for delayed cerebral ischemia, stratified by intervention type. Forest plot showing proportions of patients achieving favorable functional outcomes after endovascular treatment for delayed cerebral ischemia (DCI), stratified by intervention type. Each study is represented by a square with horizontal lines indicating 95% confidence intervals (CI). Square size is proportional to study weight in the meta-analysis. Studies are grouped by intervention type: combined treatment (spasmolysis and angioplasty), intra-arterial spasmolysis alone, and balloon angioplasty alone. Pooled estimates are shown as diamonds for each subgroup and overall. The common effect model assumes homogeneous treatment effects across studies, while the random effects model accounts for between-study heterogeneity. Study weights are calculated using the inverse variance method, where larger studies and those with more precise estimates receive greater weight. Heterogeneity statistics: I² represents the percentage of variability due to heterogeneity rather than sampling error; τ² (tau-squared) quantifies the between-study variance component in the random effects model; Q-test p-values assess statistical significance of heterogeneity. Subgroup differences were tested using chi-square tests: no statistically significant differences were found between intervention types (common effect: χ² = 4.23, *p* = 0.12; random effects: χ² = 2.17, *p* = 0.34). The vertical dashed line at 0.5 represents the null hypothesis of no treatment benefit (50% favorable outcome rate). Results favoring treatment efficacy appear to the right of this line. A total of 30 studies with 1,377 patients and 734 events were included in this analysis. CI, confidence interval; df, degrees of freedom; I^2^, I square statistic providing the percentage of variation across studies that is due to heterogeneity; τ² (tau-squared statistic representing between-study variance in the random effects meta-analysis; χ², (chi-squared) statistic refers to the test statistics for subgroup differences

We synthesized single-arm proportions of favorable functional outcome on the logit scale using inverse-variance weighting and fit both common-effect and random-effects models, reporting back-transformed proportions. These results are depicted as a forest plot in Fig. 3. Across 31 studies (*N* = 1,377), the pooled proportion was 0.56 (95% CI 0.50–0.61) under the random-effects model and 0.53 (0.50–0.55) under the common-effect model, with substantial heterogeneity (I² = 71%, τ² = 0.283, *p* < 0.01). By intervention: combined therapy (intra-arterial spasmolysis + balloon angioplasty; *N* = 692) yielded 0.57 (0.48–0.67) with I² = 78% (τ² = 0.396); intra-arterial spasmolysis alone (*N* = 444) yielded 0.50 (0.43–0.58) with I² = 57% (τ² = 0.131); balloon angioplasty alone (*N* = 241) yielded 0.60 (0.47–0.72) with I² = 74% (τ² = 0.277). Tests for subgroup differences were not statistically significant.

#### Stratified by clinical severity

**Fig. 4:**
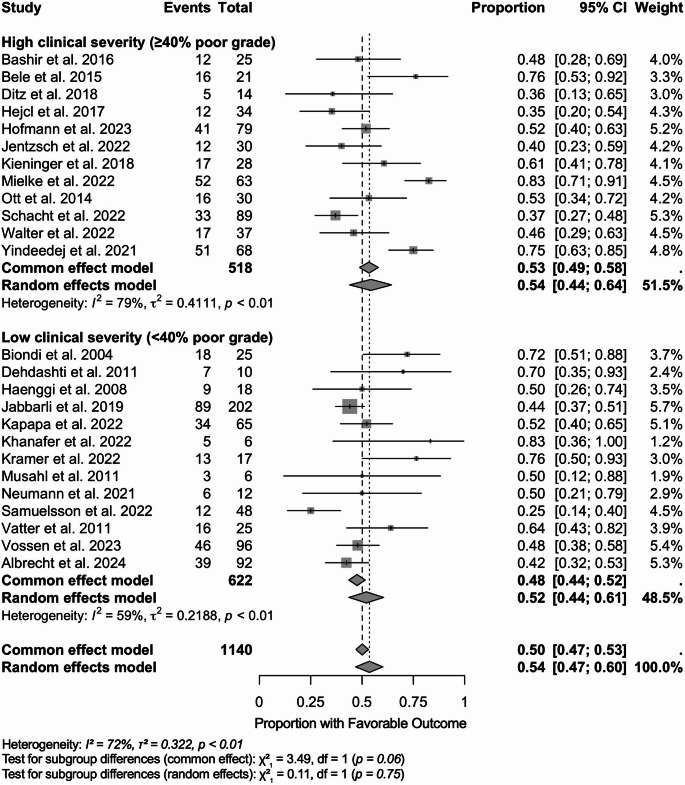
Forest plot of favorable functional outcomes following endovascular treatment for delayed cerebral ischemia, stratified by clinical severity. Forest plot showing proportions of patients achieving favorable functional outcomes after endovascular treatment for delayed cerebral ischemia (DCI), stratified by clinical severity at presentation. For this purpose, poor-grade aSAH is defined as either Hunt & Hess grade 4–5 or WFNS grade 4–5. Each study is represented by a square with horizontal lines indicating 95% confidence intervals (CI). Square size is proportional to study weight in the meta-analysis. Studies are grouped by clinical severity: high clinical severity (≥ 40% poor grade patients) and low clinical severity (< 40% poor grade patients). Pooled estimates are shown as diamonds for each subgroup and overall. The common effect model assumes homogeneous treatment effects across studies, while the random effects model accounts for between-study heterogeneity. Study weights are calculated using the inverse variance method, where larger studies and those with more precise estimates receive greater weight. Heterogeneity statistics: I² represents the percentage of variability due to heterogeneity rather than sampling error; τ² (tau-squared) quantifies the between-study variance component in the random effects model; Q-test p-values assess statistical significance of heterogeneity. Subgroup differences were tested using chi-square tests: no statistically significant differences were found between clinical severity groups (common effect: χ² = 3.49, *p* = 0.06; random effects: χ² = 0.11, *p* = 0.75). The vertical dashed line at 0.5 represents the null hypothesis of no treatment benefit (50% favorable outcome rate). Results favoring treatment efficacy appear to the right of this line. A total of 25 studies with 1,140 patients were included in this analysis. Both high and low clinical severity groups demonstrated similar treatment effectiveness, suggesting that endovascular intervention benefits patients regardless of initial clinical severity. CI, confidence interval; df, degrees of freedom; I^2^, I square statistic providing the percentage of variation across studies that is due to heterogeneity; τ² (tau-squared statistic representing between-study variance in the random effects meta-analysis; χ², (chi-squared) statistic refers to the test statistics for subgroup differences

We synthesized single-arm proportions of favorable functional outcome on the logit scale using inverse-variance weighting and fit both common-effect and random-effects models, reporting back-transformed estimates. These results are graphically depicted as a forest plot in Fig. 4. Twenty-six studies (*N* = 1,140) contributed. Overall, the pooled proportion was 0.54 (95% CI 0.47–0.60) under the random-effects model and 0.50 (0.47–0.53) under the common-effect model, with substantial heterogeneity (I² = 72%, τ² = 0.322, *p* < 0.01). In the high-severity subgroup (≥ 40% poor grade; *N* = 518), the pooled estimate was 0.54 (0.44–0.64) with I² = 79% (τ² = 0.411), contributing ~ 51.5% of the total weight. In the low-severity subgroup (< 40% poor grade; *N* = 622), the pooled estimate was 0.52 (0.44–0.61) with I² = 59% (τ² = 0.219). Tests for subgroup differences were not statistically significant (random-effects χ² = 0.11, *p* = 0.75; common-effect χ² = 3.49, *p* = 0.06).

Despite prespecified subgroup analyses by follow-up duration, intervention type, and clinical severity, substantial heterogeneity persisted across all strata (I² ranging from 57% to 80%), indicating that the pooled estimates should be interpreted with considerable caution and do not reliably represent a single underlying treatment effect.

## Discussion

### Summary of evidence

Taken together, retrospective data indicate that endovascular rescue therapies are frequently associated with angiographic improvement; however, consistent evidence of clinical benefit remains limited. This likely reflects heterogeneity in patient selection, treatment technique and intensity, as well as variation in outcome assessment and timing. The observational nature of most studies, along with missing data and unmatched cohorts, further constrains causal interpretation. These considerations informed our use of random-effects models, stratification by follow-up and intervention type, and a cautious interpretation of pooled results.

Across 39 studies including over 1,600 patients, designs, case mix, interventions, and outcome timing varied widely. Most reports were retrospective, single-center cohorts, and only two small randomized trials provided conflicting short-term results with limited follow-up. Angiographic improvement after endovascular rescue was common, but translation into infarct prevention and durable functional recovery remained inconsistent.

Although DCI-related infarction was one of the principal outcomes of interest, heterogeneous definitions and incomplete reporting precluded meaningful pooling or comparison across studies. Consequently, our quantitative synthesis focused on functional outcomes, which showed favorable recovery in roughly half of treated patients, albeit with substantial heterogeneity. Stratified analyses by follow-up duration, intervention type, and clinical severity did not identify clear subgroup differences.

Notably, studies employing continuous intra-arterial nimodipine often reported short-term neurological improvement and, in some cases, lower infarction rates compared with conservatively treated cohorts; however, these observations were derived mainly from non-randomized data and remain susceptible to selection and indication biases. Two randomized controlled trials were included. One reported no benefit, and even a potential signal toward harm, with early endovascular rescue therapy [48]. The other suggested a reduction in stroke burden and improvement in short-term neurological outcomes, albeit with limitations in follow-up and patient selection [11]. The divergent findings of these two trials likely reflect important differences in study design, patient selection, and concomitant medical management. In the Vatter et al. trial, endovascular therapy was initiated without prior induced hypertension, a departure from standard stepwise DCI management, potentially positioning endovascular treatment as a first-line substitute rather than a true rescue strategy [48]. This may have exposed patients to procedural risk without the incremental benefit expected in a refractory setting. In contrast, Yindeedej et al. did not specify background hemodynamic management, making it difficult to determine whether short-term neurological improvements reflected a genuine treatment effect or more intensive overall care [11]. These observations suggest that endovascular rescue is most plausibly beneficial in patients who have failed adequate medical optimization, and that timing, concomitant hemodynamic management, and patient selection are likely important effect modifiers that future trials should explicitly address.

Across studies with control groups (*n* = 13), results were mixed. The majority of studies did not provide a control group with best medical treatment alone. Some studies that did, demonstrated clinical benefit while others showed comparable or inferior outcomes compared to medical therapy.

The risk of complications varied across studies. While IAN was generally associated with a favorable safety profile, balloon angioplasty carried a higher rate of procedural complications, including arterial dissection and thromboembolism. Reported complication rates ranged from 3% to 17%, with higher rates associated with mechanical interventions or repeated procedures.

Our findings are amidst prior reviews with different emphases. Ma et al. compared endovascular therapy with standard care and reported lower in-hospital mortality, but no improvement in discharge or follow-up functional outcomes and longer ICU and hospital stays [49]. Our single-arm proportions are consistent with uncertain functional benefit at follow-up yet cannot address mortality. Boulouis et al., pooling broader CVS-targeted strategies, suggested a relative benefit of intra-arterial approaches in refractory vasospasm [50]; In this review differences in scope, comparators, and timeframe likely explain the discrepancy with our non-comparative, function-focused estimates. Viderman et al. summarized adverse effects of continuous intra-arterial nimodipine, highlighting hypotension and hematologic complications; this aligns with safety signals noted in several cohorts in our review, although we did not meta-analyze harms [51].

Taken together, the persistent heterogeneity across all subgroup strata likely reflects a combination of clinical and methodological sources rather than a uniform treatment effect, and the pooled summary estimates should therefore be interpreted cautiously rather than as precise point estimates of treatment efficacy.

#### Limitations

Evidence quality was limited. Most studies were observational, small, and single-center, often without concurrent controls, leaving results prone to selection, confounding, and information biases. Outcome scales (mRS vs. GOS) and assessment time points varied; despite stratification, clinical and methodological heterogeneity remained high. A further source of heterogeneity relates to the diagnostic criteria used to define the treated condition. Whereas current consensus defines DCI as a clinical syndrome encompassing new neurological deterioration not explained by other causes, several included studies used angiographic vasospasm alone as the indication for endovascular treatment, irrespective of clinical symptoms. This may have led to the inclusion of patients without true ischemic compromise, potentially diluting the observed treatment effect on functional outcomes and complicating interpretation of pooled results. Our primary synthesis used single-arm proportions, which describe outcomes after rescue therapy but cannot establish comparative effectiveness versus medical management or optimal treatment sequencing. Reporting of angiographic endpoints, perfusion metrics, and DCI-related infarction was inconsistent, precluding quantitative pooling for these outcomes. Safety reporting was also heterogeneous in definition, ascertainment, completeness, and denominator (patient *versus* intervention level), precluding meta-analysis of adverse events. Complication rates are therefore presented narratively and in a stratified supplementary table. Among pharmacologic spasmolysis studies, the most frequently reported adverse events were systemic hypotension and access-site complications, with generally low rates, though one outlying series reported adverse events in up to 75% of patients [25]. Among studies employing mechanical angioplasty or combined approaches, vessel dissection and thromboembolic events were more commonly reported, with procedural complication rates ranging from 1.2% to 25%.

In general, sources of heterogeneity in this review are likely multifactorial. Key contributors, apart from those mentioned above, include differences in patient case mix and baseline severity, heterogeneity in the type, dose, and timing of endovascular intervention, inconsistent use of concomitant medical therapies such as induced hypertension, and the assessment of outcome at variable time points. The mix of study designs, ranging from small single-center case series to larger retrospective cohorts and two small randomized trials, further amplifies between-study variance. Although prespecified subgroup analyses by follow-up duration, intervention type, and clinical severity were performed to explore these sources, none identified statistically significant differences, likely reflecting insufficient power within subgroups rather than true homogeneity of effect.

Risk-of-bias assessment (mean NOS 5.15/9) indicated generally moderate to high risk, with frequent shortcomings in group comparability and follow-up. Finally, intervention categorization was not uniform across studies, and some cohorts combined pharmacologic and mechanical rescue, limiting inference about the relative contribution of specific techniques.

## Conclusion

Across 39 studies, endovascular rescue for delayed cerebral ischemia after aneurysmal subarachnoid hemorrhage was consistently associated with angiographic improvement, yet its impact on functional recovery remained inconsistent. Pharmacologic intra-arterial spasmolysis was generally associated with a favorable safety profile, with systemic hypotension and access-site complications being the most frequently reported events. Mechanical angioplasty carried higher procedural risks, including vessel dissection and thromboembolism, and warrants careful patient selection. Direct comparison of complication rates across studies is limited by inconsistent reporting practices, including variation in whether adverse events were quantified per patient or per procedure.

Current evidence does not establish superiority over medical management. Future research should prioritize adequately powered, multicenter randomized trials; standardized criteria for patient selection, timing, and outcome assessment; and transparent reporting of adverse events. Until such data are available, endovascular treatment should be regarded as a rescue option for carefully selected patients, acknowledging the ongoing uncertainty regarding its comparative clinical effectiveness.

## Supplementary Information

Below is the link to the electronic supplementary material.

Supplementary Material 1

## Data Availability

No datasets were generated or analysed during the current study.

## Abbreviations

AHRQ: Agency for Healthcare Research and Quality
aSAH: Aneurysmal subarachnoid hemorrhage
cs: case series
CI: Confidence interval
CT: Computed tomography
DCI: Delayed cerebral ischemia
GLMM: Generalized linear mixed effects model
GOS: Glasgow outcome scale
ICU: intensive care unit
mRS: modified Ranking scale
NOS: Newcastle–Ottawa Scale
p-co: Prospective cohort study
r-co: Retrospective cohort study
RCT: randomized controlled trial
RoB: Risk of bias
