# Correction to: Endovascular treatment of symptomatic vasospasm and delayed cerebral ischemia after aneurysmal subarachnoid hemorrhage – a systematic review and meta-analysis

**DOI:** 10.1007/s00234-026-04082-w

**Published:** 2026-06-25

**Authors:** Michael Veldeman, Tobias Rossmann, Roel Haeren, Hanna Schenck, Rahul Raj, Charlotte S. Weyland

**Affiliations:** 1 Department of Neurosurgery, Universitätsklinikum Aachen, Aachen, Germany; 2 Department of Neurosurgery, Johannes Kepler University of Linz, Linz, Austria; 3 Department of Neurosurgery, Maastricht University Medical Centre, Maastricht, Netherlands; 4 Department of Neurosurgery, Helsinki University Hospital, Helsinki, Finland; 5 Department of Diagnostic and Interventional Neuroradiology, Universitätsklinikum Aachen, Aachen, Germany

**Correction to: Neuroradiology (2026) 68:1103–1116**

10.1007/s00234-026-03986-x

The original version of this article was revised.

The author’s name Hanna Schenck was incorrectly written Hanna Schenk with missing ‘c.

The original article has been corrected.

