# Supplementary material for: Endovascular treatment of symptomatic vasospasm and delayed cerebral ischemia after aneurysmal subarachnoid hemorrhage – a systematic review and meta-analysis
Source: Neuroradiology. 2026 Mar 31;68(4):1103–16. doi: 10.1007/s00234-026-03986-x (PMC13139267; doi:10.1007/s00234-026-03986-x)
Supplement: Supplementary file 1 — Supplementary Material 1 [file 234_2026_3986_MOESM1_ESM.docx]

**Supplemental Table 1**

| **Study - Year (Country)** | **Design** | **N (ERT)** | **Control Group** | **Intervention** | **Spasmolytic Agent** | **DCI Definition** | **Aneurysm Clipped (%)** | **Outcome Measure** | **Max Follow-up** | **Favorable Outcome (%)** | **Clinical Grade Tool** | **Poor Grade aSAH (%)** | **Radiological Grade Tool** | **Severe Radiological Grade (%)** |
| --- | --- | --- | --- | --- | --- | --- | --- | --- | --- | --- | --- | --- | --- | --- |
| **Adami et al. 2019 (Germany)** | **r-co** | 88 | No | Both | Not specified | Clinical, angiographic | 43% | mRS | 3 mo | Not reported | HH | 34% | Not specified | Not reported |
| **Albrecht et al. 2024 (Germany)** | **r-co** | 92 | Yes | TBA | Nimodipine | Clinical, TCD | Not reported | mRS | Not reported | 43% | HH | 36% | mFisher | 88% |
| **Andereggen et al. 2017 (Switzerland)** | **r-co** | 83 | No | Both | Not specified | Angiographic, INM | 29% | mRS | 5 mo | 60% | HH | Not reported | Fisher | Not reported |
| **Anthofer et al. 2022 (Germany)** | **r-co** | 64 | Yes | IAS | Nimodipine | Angiographic | 33% | Other | Not reported | Not reported | HH | 44% | Fisher | 100% |
| **Bashir et al. 2016 (Denmark)** | **cs** | 25 | No | IAS | Nimodipine | Clinical, angiographic | 33% | mRS | 3 mo | 48% | WFNS | 40% | Fisher | 40% |
| **Bele et al. 2015 (Germany)** | **cs** | 21 | Yes | IAS | Nimodipine | Clinical, TCD | 45% | GOS | 6 mo | 76% | WFNS | 48% | Fisher | 43% |
| **Biondi et al. 2004 (France)** | **cs** | 25 | No | IAS | Nimodipine | Clinical, angiographic | 39% | GOS | 6 mo | 72% | WFNS | 28% | Fisher | 92% |
| **Cho et al. 2011 (Korea)** | **cs** | 42 | No | IAS | Nimodipine | Clinical, angiographic | 29% | GOS | 6 mo | Not reported | HH | 12% | Fisher | 83% |
| **Coenen et al. 1998 (USA)** | **cs** | 69 | Yes | Both | Papaverine | Not defined | 100% | GCS | 0 mo | 71% | Not specified | Not reported | Not specified | Not reported |
| **Dehdashti et al. 2011 (Switzerland)** | **r-co** | 10 | No | Both | Nimodipine, verapamil | Not defined | 70% | GOS | 12 mo | 70% | WFNS | 20% | Fisher | 80% |
| **Ditz et al. 2018 (Germany)** | **r-co** | 14 | Yes | Both | Nimodipine | Clinical, TCD | 27% | mRS | 6 mo | 36% | WFNS | 67% | Fisher | 92% |
| **Duman et al. 2017 (Turkey)** | **r-co** | 25 | No | IAS | Nimodipine, milrinone | Clinical, angiographic | 0% | mRS | 18 mo | Not reported | WFNS | Not reported | Fisher | Not reported |
| **Goel et al. 2016 (India)** | **r-co** | 39 | Yes | IAS | Nimodipine | Clinical, angiographic | 100% | mRS, GOS | 3 mo | 59% | Not specified | Not reported | Not specified | Not reported |
| **Haenggi et al. 2008 (Germany)** | **p-co** | 18 | No | Both | Nimodipine | Angiographic, TCD | 58% | mRS | 3 mo | 50% | WFNS | 32% | Fisher | 85% |
| **Hejcl et al. 2017 (Czech Republic)** | **r-co** | 34 | No | IAS | Nimodipine, milrinone | TCD, CTA | 82% | GOS | 12 mo | 35% | HH | 50% | Fisher | Not reported |
| **Hofmann et al. 2023 (Germany)** | **r-co** | 79 | No | IAS | Nimodipine | Clinical, CTP | 50% | mRS | 6 mo | 52% | WFNS | 42% | Fisher | 78% |
| **Hosmann et al. 2020 (Austria)** | **p-co** | 10 | No | IAS | Papaverine | INM | 20% | mRS | 3 mo | Not reported | Not specified | Not reported | Not specified | Not reported |
| **Jabbarli et al. 2019 (Germany)** | **r-co** | 202 | Yes | Both | Nimodipine | Clinical, TCD | 37% | mRS | 6 mo | 44% | HH | 35% | Fisher | 84% |
| **Jentzsch et al. 2022 (Germany)** | **r-co** | 30 | Yes | IAS | Nimodipine, milrinone | Clinical, TCD | 15% | mRS | 6 mo | 40% | WFNS | 47% | Fisher | 93% |
| **Kapapa et al. 2022 (Germany)** | **r-co** | 65 | No | IAS | Nimodipine | CTP, CTA | 25% | GOS | 6 mo | 53% | HH | 37% | Fisher | 92% |
| **Kerz et al. 2012 (Germany)** | **r-co** | 30 | No | TBA | Nimodipine, papaverine | Clinical, angiographic | 46% | mRS | Not reported | Not reported | HH | Not reported | Fisher | Not reported |
| **Khanafer et al. 2022 (Germany)** | **r-co** | 6 | Yes | Both | Nimodipine, milrinone | Not defined | Not reported | mRS | 6 mo | 83% | HH | 33% | Fisher | 83% |
| **Kieninger et al. 2018 (Germany)** | **r-co** | 28 | No | TBA | Nimodipine | Angiographic, TCD | 11% | GOS | 6 mo | 61% | WFNS | 43% | Not specified | Not reported |
| **Kramer et al. 2022 (Germany)** | **r-co** | 17 | No | TBA | Nimodipine | TCD, CTP | 26% | GOS | 12 mo | 77% | HH | 35% | Fisher | 100% |
| **Kwon et al. 2019 (Korea)** | **r-co** | 12 | No | Both | Nimodipine, nicardipine | Clinical | 50% | mRS | Not reported | 58% | Not specified | Not reported | Not specified | Not reported |
| **Mielke et al. 2022 (Germany)** | **r-co** | 63 | Yes | Both | Nimodipine | Clinical, angiographic | 53% | mRS | 3 mo | 83% | WFNS | 44% | Fisher | 97% |
| **Musahl et al. 2011 (Germany)** | **r-co** | 6 | No | TBA | Nimodipine | Angiographic | 33% | mRS | 3 mo | 50% | WFNS | 33% | Fisher | 83% |
| **Neumann et al. 2021 (Germany)** | **r-co** | 12 | No | Both | Nimodipine | Clinical, TCD | 0% | mRS | 3 mo | 50% | WFNS | 33% | Fisher | 92% |
| **Ott et al. 2014 (Germany)** | **r-co** | 30 | No | TBA | Nimodipine | Angiographic, CTP | 97% | GOS | Not reported | 53% | HH | 40% | Fisher | 73% |
| **Samuelsson et al. 2022 (Sweden)** | **r-co** | 48 | No | Both | Nimodipine | Clinical, TCD | 19% | mRS | 6 mo | 25% | WFNS | 25% | Fisher | 90% |
| **Schacht et al. 2022 (Germany)** | **r-co** | 89 | No | IAS | Nimodipine | Clinical, TCD | 30% | mRS | 3 mo | 37% | WFNS | 44% | Fisher | 87% |
| **Stiefel et al. 2006 (USA)** | **r-co** | 5 | No | Both | Papaverine | Clinical, TCD | Not reported | Other | Not reported | Not reported | HH | 100% | Not specified | Not reported |
| **Vatter et al. 2011 (Germany)** | **p-co** | 25 | No | Both | Nimodipine | Clinical, angiographic | 42% | mRS, KPI | 6 mo | 64% | HH | 28% | Fisher | 100% |
| **Vatter et al. 2022 (Germany)** | **rct** | 34 | Yes | Both | Nimodipine | MR perfusion | 50% | mRS | 6 mo | 70% | WFNS | Not reported | Fisher | Not reported |
| **Vossen et al. 2023 (Germany)** | **p-co** | 96 | No | Both | Nimodipine | Clinical, TCD | 44% | mRS | 12 mo | 48% | HH | 29% | mFisher | 74% |
| **Walter et al. 2022 (Germany)** | **r-co** | 37 | Yes | IAS | Nimodipine | Clinical, TCD | 50% | mRS | 24 mo | 46% | HH | 54% | mFisher | 100% |
| **Weiss et al. 2019 (Germany)** | **p-co** | 33 | No | Both | Nimodipine | Clinical, TCD | Not reported | mRS | Not reported | Not reported | Not specified | Not reported | Not specified | Not reported |
| **Yindeedej et al. 2021 (Thailand)** | **rct** | 68 | Yes | TBA | Nimodipine | Angiographic | 74% | mRS | Not reported | 75% | WFNS | 40% | mFisher | 93% |
| **Zaeske et al. 2024 (Germany)** | **r-co** | 19 | No | Both | Nimodipine | TCD, CTP | 37% | Other | Not reported | Not reported | HH | 58% | mFisher | 95% |

**Supplementary Table 1: Characteristics of Included Studies**

Summary of study characteristics, patient populations, interventions, and outcomes for the 39 studies included in the systematic review (38 contributing to meta-analysis). Sample sizes reflect only patients who received endovascular rescue treatment (ERT) for delayed cerebral ischemia (DCI). Control group patients were excluded from the pooled analysis due to heterogeneous definitions across studies.

**Favorable functional outcome:** defined as mRS 0–2 or GOS 4–5 at the reported follow-up time point.

**Poor clinical grade:** proportion of patients presenting with Hunt & Hess grade 4–5 or WFNS grade 4–5 at admission.

**Severe radiological grade:** proportion of patients with Fisher grade 3–4 or modified Fisher grade 3–4 on initial imaging. Pooling of these scales is acknowledged as a limitation.

**Aneurysm clipped (%):** percentage of aneurysms secured by surgical clipping. Endovascular aneurysm treatment encompasses diverse techniques (coiling, flow diversion, intrasaccular devices) and was not summarized as a single measure.

**Study design color coding:** rct (green) = randomized controlled trial; p-co (blue) = prospective cohort; r-co (grey) = retrospective cohort; cs (amber) = case series.

cs, case series; CTA, computed tomography angiography; CTP, computed tomography perfusion; ERT, endovascular rescue treatment; Fisher, Fisher grading scale; GCS, Glasgow Coma Scale; GOS, Glasgow Outcome Scale; HH, Hunt & Hess grading; IAS, intra-arterial spasmolysis; INM, invasive neuromonitoring; KPI, Karnofsky Performance Index; mo, months; mFisher, modified Fisher grading scale; mRS, modified Rankin Scale; MR perfusion, magnetic resonance perfusion; p-co, prospective cohort study; r-co, retrospective cohort study; rct, randomized controlled trial; aSAH, aneurysmal subarachnoid hemorrhage; TBA, transluminal balloon angioplasty; TCD, transcranial Doppler sonography; WFNS, World Federation of Neurosurgical Societies grading.

**Supplementary Table 2**

| **Study (Year)** | **Representativeness** | **Selection Non-Exposed** | **Ascertainment of Exposure** | **Outcome Not Present at Start** | **Comparability** | **Outcome Assessment** | **Length of Follow-up** | **Adequacy of Follow-up** | **NOS Score** | **AHRQ Rating** |
| --- | --- | --- | --- | --- | --- | --- | --- | --- | --- | --- |
| **Adami et al. 2019** | ☆ | ☆ | ☆ | ★ | ☆ | ★ | ★ | ☆ | **3** | **Poor** |
| **Albrecht et al. 2024** | ★ | ★ | ★ | ★ | ☆ | ★ | ★ | ★ | **7** | **High** |
| **Andereggen et al. 2017** | ★ | ★ | ☆ | ★ | ☆ | ★ | ★ | ★ | **6** | **Moderate** |
| **Anthofer et al. 2022** | ★ | ★ | ★ | ★ | ☆ | ☆ | ☆ | ★ | **5** | **Moderate** |
| **Bashir et al. 2016** | ☆ | ☆ | ★ | ★ | ☆ | ★ | ★ | ★ | **5** | **Moderate** |
| **Bele et al. 2015** | ☆ | ☆ | ★ | ★ | ☆ | ★ | ★ | ☆ | **4** | **Poor** |
| **Biondi et al. 2004** | ★ | ★ | ★ | ★ | ☆ | ★ | ★ | ☆ | **6** | **Moderate** |
| **Cho et al. 2011** | ★ | ★ | ★ | ★ | ☆ | ★ | ★ | ☆ | **6** | **Moderate** |
| **Coenen et al. 1998** | ★ | ☆ | ★ | ★ | ☆ | ★ | ☆ | ☆ | **4** | **Poor** |
| **Dehdashti et al. 2011** | ☆ | ★ | ☆ | ★ | ☆ | ★ | ★ | ☆ | **4** | **Poor** |
| **Ditz et al. 2018** | ★ | ★ | ★ | ★ | ★ | ★ | ★ | ☆ | **7** | **High** |
| **Duman et al. 2017** | ★ | ☆ | ★ | ★ | ☆ | ★ | ★ | ☆ | **5** | **Moderate** |
| **Goel et al. 2016** | ★ | ★ | ★ | ★ | ☆ | ★ | ★ | ☆ | **6** | **Moderate** |
| **Haenggi et al. 2008** | ★ | ★ | ★ | ★ | ☆ | ★ | ★ | ☆ | **6** | **Moderate** |
| **Hejcl et al. 2017** | ★ | ☆ | ★ | ★ | ☆ | ★ | ★ | ☆ | **5** | **Moderate** |
| **Hofmann et al. 2023** | ★ | ★ | ★ | ★ | ☆ | ★ | ★ | ☆ | **6** | **Moderate** |
| **Hosmann et al. 2020** | ★ | ☆ | ★ | ★ | ☆ | ★ | ★ | ☆ | **5** | **Moderate** |
| **Jabbarli et al. 2019** | ★ | ★ | ★ | ★ | ★ | ★ | ★ | ★ | **8** | **High** |
| **Jentzsch et al. 2022** | ☆ | ☆ | ★ | ★ | ☆ | ★ | ★ | ★ | **5** | **Moderate** |
| **Kapapa et al. 2022** | ★ | ★ | ★ | ★ | ☆ | ★ | ★ | ★ | **7** | **High** |
| **Kerz et al. 2012** | ★ | ☆ | ★ | ★ | ☆ | ☆ | ☆ | ☆ | **3** | **Poor** |
| **Khanafer et al. 2022** | ★ | ☆ | ☆ | ★ | ☆ | ★ | ★ | ★ | **5** | **Moderate** |
| **Kieninger et al. 2018** | ★ | ☆ | ★ | ★ | ☆ | ★ | ★ | ★ | **6** | **Moderate** |
| **Kramer et al. 2022** | ★ | ★ | ★ | ★ | ☆ | ★ | ★ | ★ | **7** | **High** |
| **Kwon et al. 2019** | ☆ | ☆ | ★ | ★ | ☆ | ☆ | ☆ | ☆ | **2** | **Poor** |
| **Mielke et al. 2022** | ★ | ★ | ★ | ★ | ★ | ★ | ★ | ★ | **8** | **High** |
| **Musahl et al. 2011** | ☆ | ☆ | ★ | ★ | ☆ | ★ | ★ | ★ | **5** | **Moderate** |
| **Neumann et al. 2021** | ☆ | ☆ | ★ | ★ | ☆ | ★ | ★ | ★ | **5** | **Moderate** |
| **Ott et al. 2014** | ☆ | ☆ | ★ | ★ | ☆ | ☆ | ☆ | ☆ | **2** | **Poor** |
| **Samuelsson et al. 2022** | ☆ | ☆ | ★ | ★ | ☆ | ★ | ★ | ☆ | **4** | **Poor** |
| **Schacht et al. 2022** | ☆ | ☆ | ★ | ★ | ★ | ★ | ★ | ☆ | **5** | **Moderate** |
| **Stiefel et al. 2006** | ☆ | ☆ | ☆ | ★ | ☆ | ☆ | ☆ | ☆ | **1** | **Poor** |
| **Vatter et al. 2011** | ☆ | ★ | ★ | ★ | ☆ | ★ | ★ | ☆ | **5** | **Moderate** |
| **Vatter et al. 2022** | ★ | ★ | ★ | ★ | ★ | ★ | ★ | ★ | **8** | **High** |
| **Vossen et al. 2023** | ★ | ★ | ★ | ★ | ☆ | ★ | ★ | ★ | **7** | **High** |
| **Walter et al. 2022** | ★ | ★ | ★ | ★ | ☆ | ★ | ★ | ☆ | **6** | **Moderate** |
| **Weiss et al. 2019** | ☆ | ☆ | ★ | ★ | ☆ | ★ | ★ | ★ | **5** | **Moderate** |
| **Yindeedej et al. 2021** | ☆ | ☆ | ★ | ★ | ★ | ★ | ☆ | ☆ | **4** | **Poor** |
| **Zaeske et al. 2024** | ☆ | ★ | ★ | ★ | ☆ | ☆ | ☆ | ☆ | **3** | **Poor** |

**Supplementary Table 2: Risk of Bias Assessment (Newcastle-Ottawa Scale)**

Risk of bias assessment for all included non-randomized studies using the Newcastle-Ottawa Scale (NOS), with scores ranging from 0 to 8 across eight domains. Overall quality ratings were assigned according to Agency for Healthcare Research and Quality (AHRQ) standards: High quality (7-8 stars), Moderate quality (5-6 stars), Poor quality (0-4 stars). Stars were assigned as follows: ★ = criterion met; ☆ = criterion not met.

**Domain Definitions**

**Representativeness:** Studies reporting the total number of aSAH patients during the inclusion period and selection criteria for endovascular treatment, or describing a consecutive patient series. Either criterion sufficed for a star.

**Selection Non-Exposed:** Clear definition of endovascular treatment indications specifying patient eligibility criteria. Studies with well-described control groups or adequate baseline characteristics of the source cohort received a star.

**Ascertainment of Exposure:** Well-described endovascular vasospasm treatment protocols. Studies with vague descriptions of treatment type (spasmolysis vs. angioplasty) received no star.

**Outcome Not Present at Start:** Considered inherently satisfied for all studies, as patients with DCI represent an acute condition requiring intervention.

**Comparability:** Attempts to control for confounding variables between treatment groups. Only applicable to studies with some form of control group.

**Outcome Assessment:** Objective outcome measures with direct patient relevance. Studies reporting infarction rates and/or clinical outcomes at discharge or follow-up received a star. Angiographic resolution alone or changes in invasive neuromonitoring were insufficient.

**Length of Follow-up:** Minimum 3-month follow-up with functional outcome assessment, preferably using modified Rankin Scale. Clinical outcomes at discharge alone were insufficient.

**Adequacy of Follow-up:** Standardized follow-up methodology with documented clinical evaluation or structured telephone interviews for ordinal outcome assessment. Reconstruction from medical records alone received no star.

AHRQ, Agency for Healthcare Research and Quality; aSAH, aneurysmal subarachnoid hemorrhage; DCI, delayed cerebral ischemia; NOS, Newcastle-Ottawa Scale.

**Supplementary Table 3**

| **Study (Year)** | **N** | **Adverse Events Reported** | **Reported Adverse Events** |
| --- | --- | --- | --- |
| **Intra-arterial Spasmolysis (Pharmacologic)** | | | |
| Anthofer et al. (2022) | 64 | Yes | Hemodynamic and cardiac complications; increased infectious complications. Access-site events: 1 (1.6%) femoral artery aneurysm spurium, 1 (1.6%) femoral artery dissection, 1 (1.6%) leg arterial thrombosis. 1 (1.6%) heparin-induced thrombocytopenia. None required surgery. |
| Bashir et al. (2016) | 25 | Yes | One (4.0%): hypotension from accidental rapid nimodipine infusion. |
| Bele et al. (2015) | 21 | Yes | Two (9.5%) procedure-related microcatheter complications: 1 embolic MCA occlusion (aspirated; patient later died of multi-infarct syndrome); 1 embolic/spastic posterior communicating artery occlusion (resolved, no infarction at discharge). |
| Biondi et al. (2004) | 25 | Yes | No adverse events observed. |
| Cho et al. (2011) | 42 | Yes | Two (4.7%) asymptomatic MCA branch thromboses (1 resolved with IA tirofiban), 1 (2.4%) transient catheter-related MCA spasm. No drug-related complications. |
| Duman et al. (2017) | 25 | No | Not reported. |
| Goel et al. (2016) | 39 | Yes | One (2.6%) inguinal hematoma. Hypotension in 6 patients (15.4%) after IAN, of whom 3 (7.7%) required inotropes. |
| Hejcl et al. (2017) | 34 | Yes | Five episodes of hypotension (11.6% of 43 interventions, predominantly milrinone-related); transient clinical deterioration in 2/43 (4.7%) of interventions. |
| Hofmann et al. (2023) | 79 | No | Not reported. |
| Hosmann et al. (2020) | 10 | Yes | No adverse events observed. |
| Jentzsch et al. (2022) | 30 | Yes | No major periprocedural complications. Minor sheath dislocations or occlusions mentioned without quantification. |
| Kapapa et al. (2022) | 65 | Yes | Adverse event in 36 patients (55.4%); complication in 49 (75.4%); new DCI-related infarction in 33 (50.8%); IA spasmolysis-related mortality in 4 (6.2%); severe complications 5 (7.7%). |
| Schacht et al. (2022) | 89 | Yes | No adverse events observed. |
| Walter et al. (2022) | 37 | Yes | No adverse events observed. |
| **Intra-arterial Angioplasty (Mechanical)** | | | |
| Kerz et al. (2012) | 30 | No | Not reported. |
| Kieninger et al. (2018) | 28 | Yes | One (3.6%) intracranial hemorrhage; 4 (14.3%) cardiac arrests; 3 (10.7%) stage-I acute kidney failure; 1 (3.6%) heparin-induced thrombocytopenia. No clinically relevant thromboembolic infarctions. |
| Kramer et al. (2022) | 17 | Yes | Two (11.8%) accidental catheter removals; 1 (5.9%) temporal lobe vasogenic edema (nimodipine-related); 1 (5.9%) MCA perforation; 2 (11.8%) ICA dissections. No catheter-associated infarctions. |
| Musahl et al. (2011) | 6 | Yes | One (16.7%) intracranial hemorrhage, possibly related to systemic heparinization. |
| Ott et al. (2014) | 30 | Yes | No adverse events observed. |
| Yindeedej et al. (2021) | 68 | Yes | Reported per treatment group: 3/68 (4.4%) thromboembolic complications: 2/36 (5.6%) in the intervention group and 1/32 (3.1%) in the control group, all treated with thrombectomy *versus* 1/32 (3.1%) groin hematoma in the control group, managed conservatively. |
| Albrecht et al. (2024) | 92 | Yes | 4/92 patients (4.3%) with procedure-related complications across 241 interventions (1.7% per procedure): 1 extracranial vessel dissection, 2 intracranial vessel dissections, 1 groin pseudoaneurysm. |
| **Combined (Spasmolysis + Angioplasty)** | | | |
| Adami et al. (2019) | 88 | Yes | Thromboembolic complication or arterial dissection related to PTA: 3/20 (15.0%) of PTA procedures. |
| Andereggen et al. (2017) | 83 | Yes | Vessel dissections in 6/83 (7.2%) patients. |
| Coenen et al. (1998) | 69 | No | Not reported. |
| Dehdashti et al. (2011) | 10 | Yes | One (10.0%) procedure-related death (thromboembolic carotid bifurcation occlusion). One (10.0%) rebleeding from incompletely occluded aneurysm (authors deemed non-procedure-related). |
| Ditz et al. (2018) | 14 | Yes | 1/65 treatment sessions (1.5%): peri-interventional M3 occlusion with subsequent infarction. |
| Haenggi et al. (2008) | 18 | Yes | Hypotension in 6/18 (33.3%). 1/18 (5.6%) death from progressive intracranial pressure rise 1 day after IAN. |
| Jabbarli et al. (2019) | 202 | Yes | EVT-related complications: 2/121 (1.7%) in cohort A and 1/81 (1.2%) in cohort B. Angioplasty-related: 1 vessel rupture, 1 thromboembolic occlusion. Pharmacologic: 1 temporary tachycardia (nimodipine). |
| Khanafer et al. (2022) | 6 | Yes | One (16.7%) patient with a complication (not further specified). |
| Kwon et al. (2019) | 12 | Yes | Three (25.0%) complications; none resulted in permanent clinical sequelae (not further specified). |
| Mielke et al. (2022) | 63 | No | Not reported. |
| Neumann et al. (2021) | 12 | Yes | No adverse events observed. |
| Samuelsson et al. (2022) | 48 | Yes | Procedure-related complications in 10/48 (20.8%): 1 MCA embolic occlusion, femoral artery dissection and pseudoaneurysm, coil loosening during recatheterization (exact numbers not specified for individual event types). |
| Stiefel et al. (2006) | 5 | No | Not reported. |
| Vatter et al. (2022) [RCT] | 34 | Yes | In the 16 endovascularly treated patients: 2 (12.5%) vessel dissections, 1 (6.3%) thromboembolic event, 1 (6.3%) embolic infarction. |
| Vatter et al. (2011) | 25 | Yes | 2/25 (8.0%) iatrogenic strokes; 1/25 (4.0%) reperfusion bleeding. Three cases with complications leading to segment lesions (1 minor, 3 major). |
| Vossen et al. (2023) | 96 | Yes | 9/96 (9.4%) procedure-related complications: 2 catheter thrombi, 1 cerebral vessel dissection, 2 access vessel dissections, 3 ischemic strokes, 1 hemorrhagic transformation of ischemic stroke. |
| Weiss et al. (2019) | 33 | Yes | No adverse events observed (minor ischemic lesions noted but not classified as procedure-related complications). |
| Zaeske et al. (2024) | 19 | No | Not reported. |

**Supplementary Table 3: Adverse Events by Intervention Type**

Summary of reported adverse events across all 39 included studies, stratified by primary intervention type. Percentages are calculated using the total number of analyzed patients (N) as the denominator, except where event rates are explicitly reported per procedure or per intervention session.

* Intervention class assigned based on primary reported technique. Studies using both pharmacologic and mechanical approaches are listed under 'Combined'. Adverse event reporting was heterogeneous across studies; rates are not directly comparable across studies.

AE, adverse event; EVT, endovascular treatment; IA, intra-arterial; IAN, intra-arterial nimodipine; MCA, middle cerebral artery; IAN, intra-arterial nimodipine; ICA, internal carotid artery; ICP, intracranial pressure; DCI, delayed cerebral ischemia; PTA, percutaneous transluminal angioplasty; RCT, randomized controlled trial.

**Supplemental Figure 1**


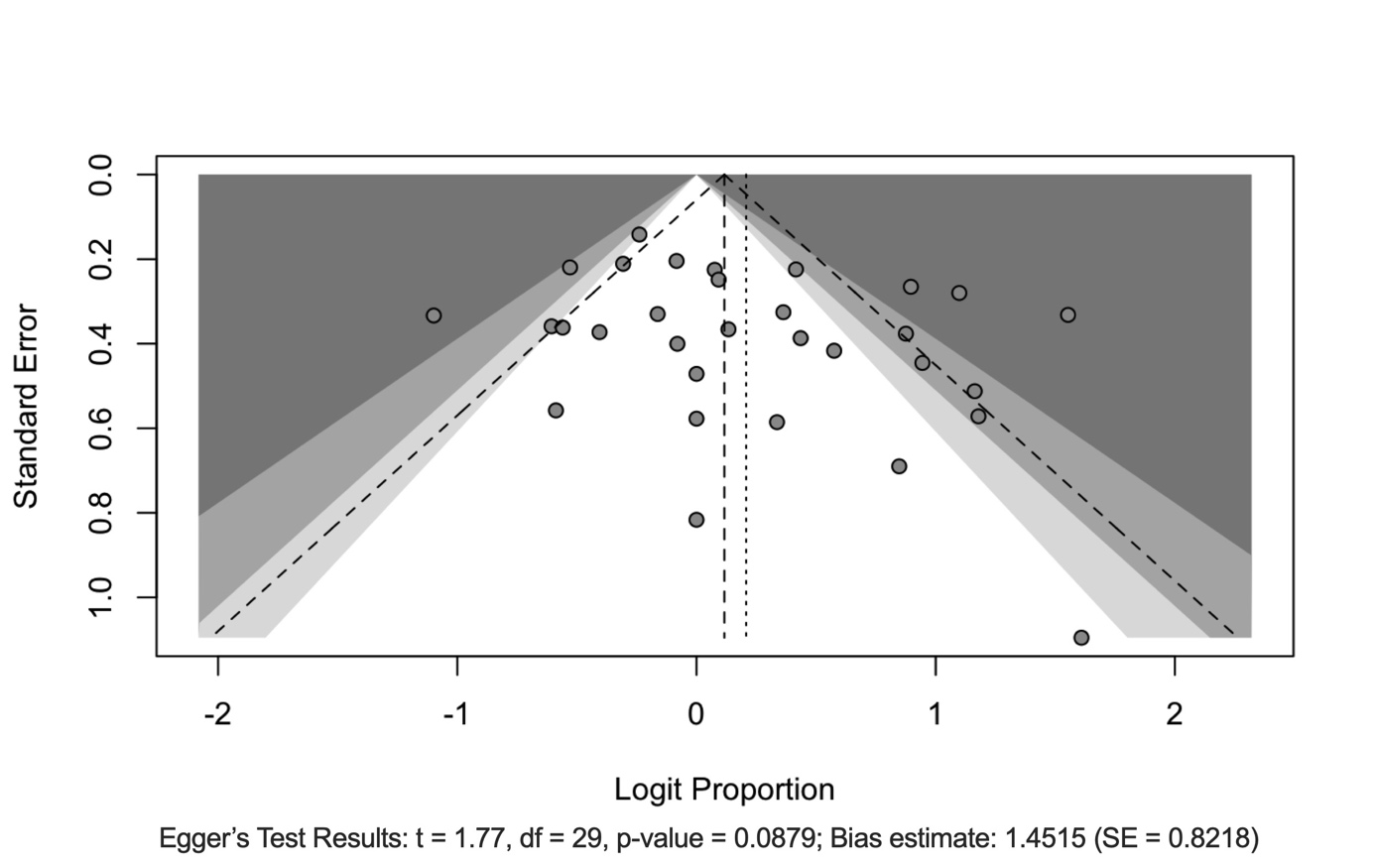


**Supplemental Figure 1.** **Funnel plot for assessment of publication bias in studies of endovascular treatment for delayed cerebral ischemia.**

Funnel plot showing the relationship between study precision (standard error) and effect size (logit-transformed proportions of favorable functional outcomes). Each circle represents an individual study. The vertical dashed line indicates the overall pooled effect estimate from the random effects meta-analysis. The diagonal dashed lines represent the 95% confidence limits around the pooled estimate, creating a triangular region where studies would be expected to fall in the absence of publication bias.

Egger's regression test was performed to statistically assess funnel plot asymmetry (t = 1.77, df = 29, p = 0.0879), indicating no statistically significant evidence of publication bias at the conventional α = 0.05 level, though the p-value approaches significance suggesting potential small-study effects.
